# Validation of the Iranian version of the Postpartum Specific Anxiety Scale 12-item research short-form for use during global crises (PSAS-IR-RSF-C)

**DOI:** 10.1186/s12888-023-04998-0

**Published:** 2023-07-14

**Authors:** Sepideh Mashayekh-Amiri, Mohammad Asghari Jafarabadi, Maryam Montazeri, Victoria Fallon, Sergio A. Silverio, Mojgan Mirghafourvand

**Affiliations:** 1 Students Research Committee, Midwifery Department, Faculty of Nursing and Midwifery, Tabriz University of Medical Sciences, Tabriz, Iran; 2 Cabrini Research, Cabrini Health, Melbourne, VIC 3144 Australia; 3 School of Public Health and Preventative Medicine, Faculty of Medicine, Nursing and Health Sciences, Monash University, Melbourne, VIC 3004 Australia; 4 Road Traffic Injury Research Center, Tabriz University of Medical Sciences, Tabriz, Iran; 5 Department of Psychology, Institute of Population Health, University of Liverpool, Liverpool, UK; 6 Department of Women and Children’s Health, School of Life Course and Population Sciences, King’s College London, London, UK; 7 Social Determinants of Health Research Center, Tabriz University of Medical Sciences, Tabriz, Iran; 8 Menopause Andropause Research Center, Ahvaz Jundishapur University of Medical Sciences, Ahvaz, Iran

**Keywords:** Global crises, Postpartum anxiety, Short-form, PSAS-IR-RSF-C, Maternal mental health, Psychometrics, Iranian women

## Abstract

**Background:**

Due to its high pervasiveness and adversarial consequences, postpartum anxiety has been one of the most worrying public health concerns in the last decade. According to previous research, the occurrence of mental disorders among women in the postpartum period upsurges significantly in the course of universal disasters. The Postpartum Specific Anxiety Scale – Research Short Form – for use in global Crises [PSAS-IR-RSF-C] has not been used in Iran for postpartum women during a health system shock. Consequently, this study was conducted to determine the validity and reliability of the Persian version (PSAS-IR-RSF-C) during the COVID-19 pandemic.

**Methods:**

This cross-sectional study was performed with 180 women who were between six weeks and six months after delivery, by random sampling method from December 2021 to June 2022. The validity of the PSAS-RSF-C in terms of face, content, was analyzed, and the construct validity was assessed using exploratory and confirmatory factor analyses. Internal consistency and test–retest reliability of the questionnaire were measured using (Cronbach’s alpha, McDonald’s ω) and intraclass correlation coefficient (ICC), respectively.

**Results:**

The content validity index and content validity ratio of the Persian version of the PSAS-IR-RSF-C were 0.96 and 0.98, respectively. A three-factor structure was extracted during the exploratory factor analysis process, and model validity was confirmed by the values of fit indices. Cronbach's alpha coefficient, McDonald’s ω and intra-cluster correlation coefficient (95% confidence interval) were 0.74, 0.92 (0.78 to 0.93) and 0.97 (0.93 to 0.98), respectively.

**Conclusions:**

For the specific assessment of postpartum anxiety among Iranian women during crises, the Persian version of the PSAS-IR-RSF-C is a valid and reliable tool.

## Background

Globally, health-related crises are the leading cause of mental disorders in all levels and age groups of society. The COVID-19 pandemic health system shock is the latest crisis with which all the countries are struggling [1]. In January 2020, the COVID-19 was declared an international public health emergency by the World Health Organization, and it did not take long for the pandemic to spread across the globe [2]. In this regard, to limit the spread of COVID-19, compliance with a series of public and social health measures, such as limiting attendance at public and private gatherings, maintaining physical distance, wearing nose and mouth masks, and quarantine, were strongly recommended by the World Health Organization [3].

Initial studies revealed poor mental health of people is associated with the various restrictions imposed by governments to slow down the spread of the virus [4, 5]. Previous studies also reveal the prevalence of mental disorders, especially among those groups thought to be vulnerable, i.e., pregnant women are significantly higher than in the general population during crises or disasters. Ignoring this can have devastating outcomes [4, 6–9].

As one of the most significant stages of a woman's life, pregnancy and childbirth offer an opportunity for a usually happy and unique experience. However, women are exposed to mental disorders like anxiety during pregnancy and the postpartum period. Pregnancy and childbirth are stressful for women, and this health crisis will double mental disorders during pregnancy and childbirth [10].

Under normal circumstances, approximately 20% of all women who give birth are thought to experience mental health problems [11]. Though the results revealed that the prevalence of postpartum anxiety increased from 18.8% in the pre-pandemic period to 34.8% in the post-pandemic period, this issue is worth considering [12].

Wide-ranging consequences such as disruption of the maternal bond, adverse outcomes of infant feeding, reduction of mothers' self-efficacy, poor outcomes of the infant's psychological development, and infant's mood problems can be related to postpartum anxiety. These problems can impose an economic burden on the health system for both the mother and the infant [13–15].

The importance of correct and timely identification of anxiety during the postpartum period, especially during global crises, is revealed by the extent and multiplicity of the mentioned consequences. Thus, to achieve this, it is essential first to identify this disorder [16–18]. There are many scales to measure postpartum anxiety (PPA), including the State-Trait Anxiety Inventory (STAI) [19], and the Generalized Anxiety Disorder-7 (GAD-7) [20]. The disadvantages of these scales are their adult population oriented design, their lack of usefulness in childbearing and their limitation on reflecting the maternal-focused concerns. Consequently, to overcome these issues, the 51-item Postpartum Specific Anxiety Scale (PSAS) was developed and validated [21]. While PSAS demonstrates good psychometric properties for measure of PPA, there are increasing requests for a shorter version to aid accessibility. Therefore, a 16-question form (PSAS-RSF) was also designed [22]. Also, a 12-question form (PSAS-RSF-C) was also developed following global requests to address mental health during crises since no tool has been available to measure postpartum anxiety during global health crises, predominantly during the COVID-19 pandemic. Silverio et al. (2021) developed the Postpartum Specific Anxiety Scale – Research Short Form – for use in global Crises [PSAS-IR-RSF-C] to cope with the lack of a suitable tool to measure mothers' anxiety quickly and accurately in times of health crises. This tool included 12 questions measured on a 4-point Likert scale. This instrument consists of four domains of anxiety relating to: maternal competence and attachment, infant safety and welfare, practical infant care, and psychological adjustment to motherhood [23].

The publication presenting the development of the 12-item tool also presented unvalidated translations into Italian, French, Chinese, Spanish, and Dutch [23]. Regarding the COVID-19 pandemic and the lack of an appropriate tool to measure maternal anxiety in Iranian speaking populations, this research sets out to translate and validate the PSAS-IR-RSF-C into Persian [PSAS-IR-RSF-C].

## Methods

### Study aim and design

This cross-sectional study was conducted to determine the psychometric properties of the Persian version of the PSAS-RSF-C. Before using the PSAS-IR-RSF-C, the required permissions were obtained from the PSAS Working Group. This study was approved by the Ethics Committee of the Tabriz University of Medical Sciences, Tabriz, Iran (ref: IR.TBZMED.REC.1400.487).

### Study population and sampling

Out of a total of 410 women, 136 women did not meet the eligibility criteria and were excluded from the study. Of the remaining 274 women who were eligible, 94 were unwilling to participate in the study. Finally, 180 women were included in the present study. The response rate was 66% (180/274). Therefore, this study was performed with 180 women who were selected using the random cluster approach. Using the website www.random.org, a quarter of Tabriz's 82 health centers were chosen for the first step of the sampling. Then, a list of mothers who were six weeks to six months postpartum (and had either given birth vaginally or via caesarean section) was extracted through the SIB system (integrated health system). The number of women chosen from each center was proportionally calculated concerning the sample size, and they were chosen at random using the same website.

The researcher then called participants using their phone number, briefly explained the goals for the study and how it would be carried out, and extended an invitation to participate. Mothers were asked to come to the health center on a specific day and time if she wanted further explanation about the study and to complete the questionnaires. Random selection of participants occurred before assessing their eligibility criteria. However, after attending the health center, participants were thoroughly evaluated for basic information and eligibility. Only those individuals who met the eligibility criteria were provided with in-depth information about the research goals, advantages, outcomes, confidentiality, and how it would be conducted and invited to participate. When a participant agreed to take part in the study, an informed consent form was filled out, and socio-demographic and obstetric characteristics checklist as well as the PSAS-IR-RSF-C were completed by them.

### Sample size determination

Factor analysis necessitates an appropriate sample size to ensure the reliability and validity of the results. We considered the guidelines provided in this regard [24], which suggest a sample size of 5 to 10 samples per instrument question. In line with these recommendations, we aimed for a sample size of 10 samples per item, given that our instrument consisted of 12 items. Therefore, a minimum of 120 responses was initially deemed necessary. However, it is important to consider the impact of the cluster sampling method employed in our study. The cluster sampling method introduces an element of intra-cluster correlation, which needs to be accounted for in the sample size calculation. To address this concern, we applied a design effect of 1.5 to adjust the sample size. As a result, the sample size was raised to 180 participants.

### Instruments

In this study two measures were used for data collection.

#### Socio-demographic and obstetric characteristics checklists

This checklist included questions about maternal age, education, occupation, income, gestational age at delivery, mode of delivery, and baby’s birth weight.

#### Persian Postpartum Specific Anxiety Scale – Research Short Form – for global Crises [PSAS-IR-RSF-C]

In the UK, Silverio et al. developed this tool in 2021 from the original 51-item PSAS. It has 12-items and four domains (three questions in each domain) of anxiety: maternal competence and attachment anxieties, infant safety and welfare anxieties, practical infant care anxieties, and psychosocial adjustment to motherhood. This 12-item self-report scale was designed to examine the frequency, and not severity, of anxieties specific to the postpartum period. The PSAS was developed and validated for use with mothers of infants aged between 0 and 6 months. The PSAS includes items about emotional distress, and anxieties related to the participant’s newborn infant. In the PSAS -RSF-C, each domain has 3 items, scored on a 4-point Likert scale (from never = 1; to almost always = 4). This questionnaire has a minimum score of 12 and a maximum score of 48. In the original scale, the four factors had good reliability, with McDonald’s ω ranging from 0.74 to 0.88. Furthermore, the overall scale had good reliability (McDonald’s ω = 0.87) [23].

### Translation procedure

The questionnaire was independently translated from English to Farsi by two translators who were native Farsi speakers and had experience in the postpartum anxiety field. Both translators were fluent in English. The discrepancies in their translation were then debated between the two translators, and the Persian version was produced after integrating the two versions and fixing the contradictions. An English-speaking translator who is conversant in Persian was then given this version to translate from Persian to English. To resolve any discrepancies between the two versions, forward–backward and the original versions were compared, and the required modifications were made [25].

### Statistical analyses

All analyses were conducted using IBM SPSS Statistics 22 (IBM Corp, Armonk, NY, USA) and STATA 14 (Statcorp, college station, Texas, USA). Data were expressed using Mean (SD) and Median (Percentile 25-Percentile75) or (min–Max) for numeric normal and non-normal variables, respectively, and frequency (percent) for categorical variables.

### Assessment of the content and face validity

The content validity ratio (CVR) and the content validity index (CVI) were calculated to verify the content's validity based on the opinions of 10 experts (specialists in midwifery and reproductive health). For each expert, a two-section checklist was created. Calculating CVI and CVR was the purpose of the first and second sections of the checklist, respectively. On a 4-point Likert scale (for instance: 1 = not relevant, 2 = relatively relevant, 3 = relevant and 4 = completely relevant), the first section of the checklist assessed the item's clarity, simplicity, and relevance to calculate the CVI. The second section used a 3-point Likert scale, from unnecessary to necessary (it is necessary; useful, but not necessary; not necessary), to assess each item's necessity to calculate CVR. Validity was defined as CVR and CVI higher than 0.62 and 0.79, respectively [26].

For the face validity, 30 eligible women completed the PSAS-IR-RSF-C and were asked to assess the questions with respect to their difficulty, appropriateness, and ambiguity and to rank the items' value on a scale of 1 (not at all important) to 5 (extremely important). Then, based on the views of women, face validity was statistically assessed using the impact score formula. The quantitative approach of item impact was employed to assess each item's importance. Using the following formula (Impact Score = Frequency (%) × Importance), the researcher determined the impact score of each question based on the answers selected by the women separately. With a score of more than 1.5, the Impact Score is accepted [27].

### Factor analysis

Exploratory factor analysis (EFA) and confirmatory factor analysis (CFA) techniques were employed to assess construct validity [28].

### Exploratory factor analysis

After computing the correlation matrix between the variables for the exploratory factor analysis, factors were extracted using the principal axis factoring method, followed by direct oblimin (to consider the relationship between factors). After the components were extracted, each one was given a name based on the variables (questions), and the degree to which these factors were compatible with the notion and dimensions of anxiety was assessed. Kaiser–Meyer–Olkin (KMO) and Bartlett's test were used to identify the number of factors and the variance index represented by each factor and the total, and the Eigenvalue technique, the scree plot diagram was also used to assess the model's suitability. The cut-off point was 0.3 for determining factor parameters [29].

### Confirmatory factor analysis

To assess the structure of the components derived from the exploratory factor analysis, confirmatory factor analysis was carried out in a separate subsample. Fit indices were used to assess how well the exploratory model fit the data. The significance of the model coefficients test and the correlation test between the components were assessed in the confirmatory factor analysis. To validate the model, Root Mean Square Approximation (RMSEA) less than 0.08, Approximation Square Mean Square Root Standardized (SRMSEA) less than 0.08, normed Chi2 < 5, Index Tucker-Lewis (TLI) ≥ 0.95, and Index Fit Index (CFI) ≥ 0.90 were taken into consideration [29].

### Reliability assessment

Test–retest reliability and internal consistency were used to assess the questionnaire's reliability. The questionnaire was filled out by 30 mothers who were chosen at random, two weeks apart, and the intra-class correlation coefficient (ICC) and its 95% confidence interval (95% CI) were calculated within the scores obtained from answering the questionnaire twice for each factor and the entire questionnaire. For each factor and the total instrument, Cronbach's alpha coefficient was utilized to assess internal consistency. Cronbach's alpha coefficient was deemed higher than 0.7 in the current study [26].

## Results

From December 2021 to June 2022, 180 mothers were randomly included in the present study from 82 health centers in Tabriz. The participants' mean age (SD) was 27.6 (5.8) years. The majority (93.9%) were housewife. The participants’ socio-demographic characteristics are given in Table 1.

**Table 1:** Socio-demographic characteristics of participants (*N* = 180)

Characteristics	N (%)
**Age** (Year)^a^	27.6 (5.8)
**Education**	
Secondary school or below	55 (30.5)
Diploma and high school	125 (69.4)
**Occupation**	
Housewife	169 (93.9)
Employee	11 (6.1)
**Income**	
Not at all sufficient	35 (19.4)
Relatively sufficient	110 (58.2)
Completely sufficient	35 (19.4)
**Gestational age** (Week)^a^	37.9 (2.0)
**Mode of delivery**	
Normal vaginal delivery (NVD)	48 (26.7)
Caesarean section (C/S)	132 (73.3)
**Baby’s weight** (Gram)^a^	3187.9 (514.8)

^a^_mean (standard deviation)_

In the content validity, all items obtained the minimum acceptable value of CVI and CVR, which were 0.96 and 0.98, respectively. Face validity indicates that all items were appropriate and without vagueness and difficulty and received a minimum impact score of 1.5 (Table 2).

**Table 2:** The impact score, CVI, and CVR for questions (*n* = 10 Expert)

Items	Impact score	CVI	CVR
1	4.00	0.86	1.00
2	4.00	0.93	1.00
3	4.00	0.83	1.00
4	4.00	1.00	1.00
5	4.00	1.00	1.00
6	4.00	1.00	1.00
7	4.00	1.00	1.00
8	4.00	1.00	1.00
9	4.00	1.00	1.00
10	4.00	0.96	0.80
11	4.00	1.00	1.00
12	4.00	1.00	1.00

*CVI* Content Validity Index, *CVR* Content Validity Ratio

**Fig. 1:**
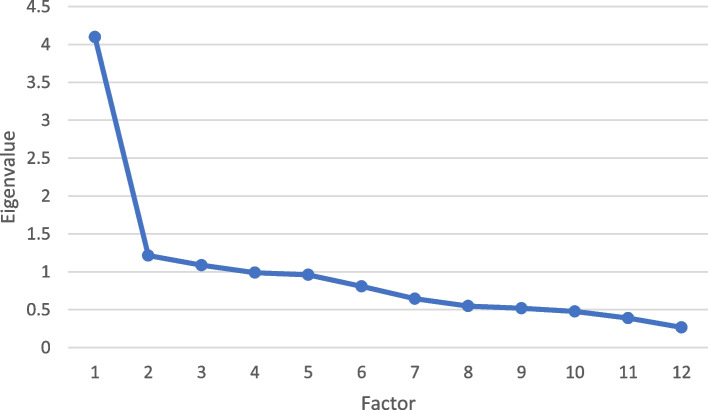
Factor load scree plot of the items for determining the number of extracted factors of the Persian version of Postpartum Specific Anxiety Scale-Research Short-Form during crisis

In the EFA process, KMO confirmed the model adequacy above 0.7 and the significant result of Bartlett's test. In the current study, Model adequacy was confirmed by KMO of 0.807 and significance of < 0.001 of the Bartlett’s test of Sphericity (Fig. 1).

According to the results of exploratory factor analysis, three factors structure was attained with a total variance of about 41.57%. The first factor includes the anxieties of practical infant care with eight questions; the second factor is the anxieties about to infant safety and welfare and includes two questions; and the third factor with two questions includes anxiety related to the marital relationship. Also, question 9 in the main scale (I felt that someone else could take care of my child better than me) was removed due to the factor load of less than 0.3 (Table 3).

**Table 3:** Facture structure of the PSAS-RSF-C

Scale item	Factor 1	Factor 2	Factor 3
**Factor 1: practical infant care anxieties**			
1. I have repeatedly checked on my sleeping baby	0.642		
2. I have worried that my baby will stop breathing while sleeping	0.599		
3. I have worried about my baby being accidentally harmed by someone or something else	0.522		
4. I have felt unconfident or incapable of meeting my baby’s basic care needs	0.511		
5. I have worried about getting my baby into a routine	0.509		
6. I have felt tired even after a good amount of rest	0.479		
7. I have had negative thoughts about the relationship with my baby	0.352		
	0.161		
**Factor 2: nutrition and growth anxieties**			
8. I have worried about my baby’s weight		-0.952	
9. I have worried about my baby’s milk intake		-0.638	
**Factor 3: marital relationship anxieties**			
10. I have worried more about my relationship with my partner than before my baby was born			0.787
12. I have felt resentment towards my partner			0.412
% Variance Explained	29.86	6.57	5.14
Total score	41.57		
Cronbach’s alpha	0.60	0.71	0.55
Total score	0.74		
McDonald’s ω	0.77 (0.71, 0.82)	0.81 (0.72, 0.88)	0.64 (0.47, 0.75)
Total score		0.92 (0.78, 0.93)	
Intraclass Correlation Coefficient (95% CI)	0.95 (0.90 to 0.98)	0.94 (0.87 to 0.97)	0.80 (0.58 to 0.90)
Total score	0.97 (0.93 to 0.98)		
Mean (SD)	5.9 (4.2)	1.7 (1.9)	1.5 (1.7)
Total score	9.1 (6.3)		

**Fig. 2:**
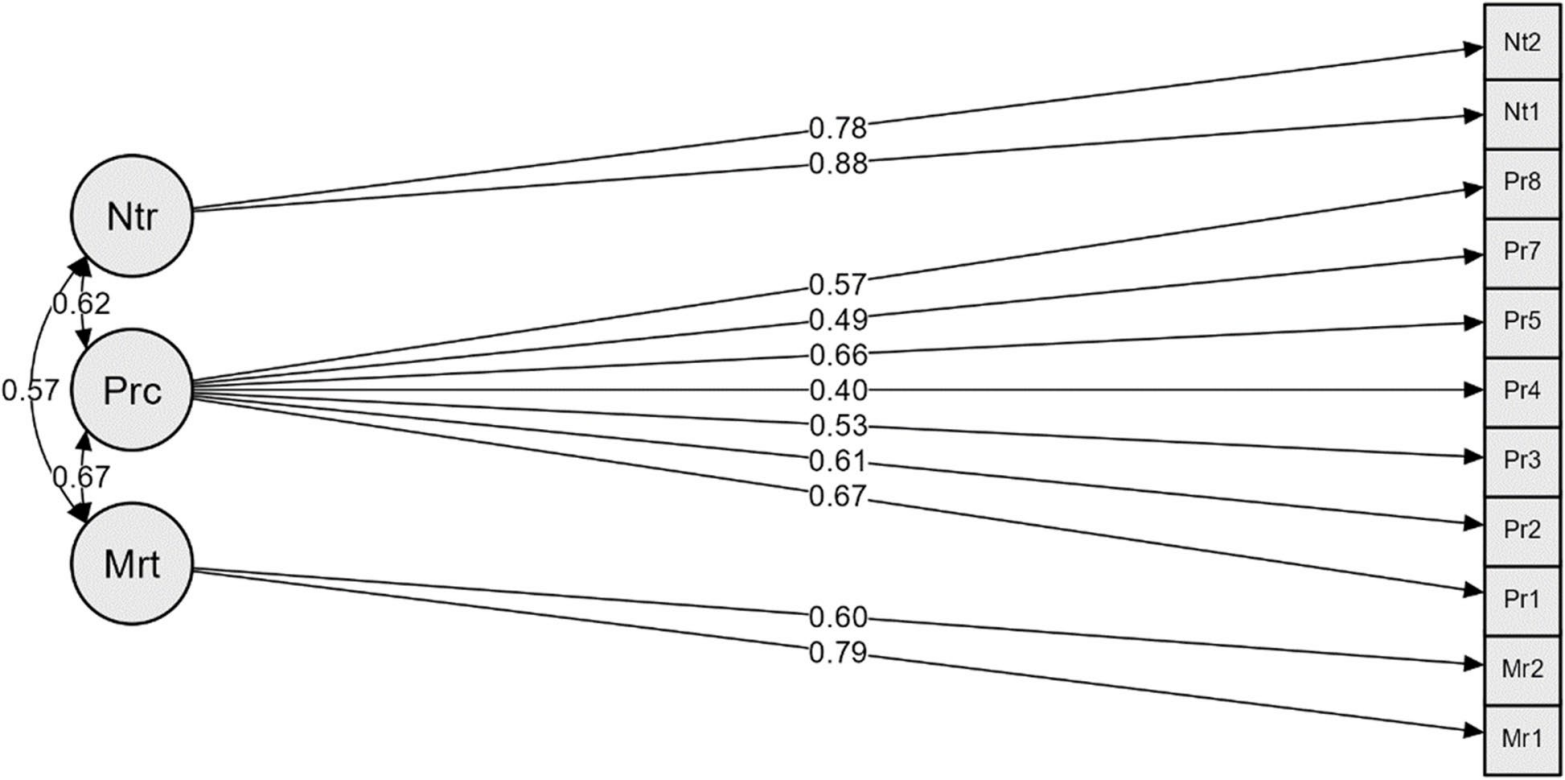
Factor structure model of the PSAS-RSF-C based on CFA. All factor-item relationships were significant (*P* < 0.05). Prc: *practical infant care anxieties, Ntr: nutrition and growth anxieties, Mrt: marital relationship anxieties*

In confirmatory factor analysis, × 2/df was 1.725, and the RMSEA index value was 0.063 (0.037, 0.088). These values confirmed the model's validity. TLI and CFI fitting indices were more significant than 0.9 (Table 4). As a result, this model has achieved a good level of fit, based on which it’s factorial structure can be confirmed, besides all factor-item relationships were significant (Fig. 2).

**Table 4:** Confirmatory factor analyses fit Index of the PSAS-RSF-C (*n* = 180)

Fit Indices	Fit
χ2	70.748
P	0.003
${}^{{x}^{2}}\!\left/ \!{}_{df}\right.$	1.725
CFI	0.943
SRMR	0.049
TLI	0.923
RMSEA (90% CI)	0.063 (0.037, 0.088)

χ2 chi-square; df degrees of freedom; χ2/df normed chi-square

*CFI* Comparative Fit Index, *SRMR* Standardized root mean squared residual, *TLI* Tucker–Lewis index, *RMSEA* Root mean square error of approximation, *CI* Confidence interval

In the test–retest, the ICC (95% CI), Cronbach's alpha coefficient and McDonald’s ω were 0.97 (0.93 to 0.98),0.74 and 0.92 (0.78 to 0.93), respectively. This indicated the good internal consistency of the questionnaire (Table 3).

## Discussion

The COVID-19 pandemic has imposed a substantial psychological burden on society, especially on postpartum women. Based on the fact that women during the postpartum period may experience conflicting emotions due to the joy of waiting for the birth of a baby, to fear of the birth process, and various anxieties linked to parenthood [30, 31], it is no wonder that restrictions created to prevent the transmission of a global pandemic, as well as the fear of infection from the virus itself in the current health system shock, may have caused postpartum women to experience more anxiety and worries than before [4–6]. Furthermore, identifying postpartum anxieties in the current health crisis is essential and requires a valid and reliable tool to measure postpartum anxiety.

The present study aimed to do a psychometric assessment of a specific short-form postpartum anxiety questionnaire during a global health crisis, in Iranian women. The results indicate that the Persian version of this scale is a valid and reliable tool for evaluating postpartum anxiety among Iranian women. Previously, a 51-question postpartum anxiety questionnaire was developed by Fallon et al. [21] in 2016, which has been translated into Persian after validation in an Iranian population [32]. Due to repeated requests to create a shorter form, a shortened form of 16 questions [22] was designed in 2021, which received much attention. After that, a 12-question version of this questionnaire was created to be used in times of crisis [23].

In order to determine the psychometric properties of this tool, its validity was determined using content validity, face validity, and construct validity. The reliability of the tool was checked and confirmed through internal consistency and test-retesting. Cronbach's alpha coefficient obtained for the scale was 0.74, which indicates its good internal consistency. In this regard, Silverio et al.'s study reported reliability for the whole questionnaire as 0.87 and its four factors in the range of (0.74–0.88) [23].

During the process of exploratory factor analysis, a 3-factor structure was obtained for 11 questions of the scale compared to its original version, which had four factors and 12 questions. Furthermore, the explained variance of the factors for measuring the desired concept in the questionnaire was about 42% (3 factors) for the 3-factor structure, which was equal to 0.75% (4 factors) in the original questionnaire [19]. Also, the value of KMO and the significance of Bartlett's test, and the value of RMSEA, 0.064, confirmed the model adequacy.

The first factor obtained during exploratory factor analysis is the anxieties about the practical infant care, which includes eight items. The second factor expresses the anxieties of infant nutrition and growth, which includes two items; the third factor concerns the anxieties caused by marital relationships, which includes two items.

Based on the results of the studies, part of the anxiety women suffer in the postpartum period is caused by anxiety related to the health and practical care of the baby. In this regard, via a structured interview, Affonso et al. investigated the effects of postpartum anxiety on 84 primiparous women and 137 multiparous women six weeks after childbirth. The most common reported theme for both groups in this study was concern about health and infant care [33].

Feeding behaviors and infant growth are other essential issues of postpartum anxiety. Generally, the first three months after delivery is a critical period for infants. The mother's mental state affects the infant's feeding behaviors during this period [34]. In this regard, a prospective cohort study disclosed that postpartum anxiety increases breastfeeding cessation in fewer than six weeks by 157% and breastfeeding for fewer than eight months by 124% [35]. Sun et al. (2020) indicated that maternal anxiety during postpartum was associated with harmful infant feeding practices, such as bottle feeding and refusal to feed the infant [36].

Consequently, the mother's mental health may be an essential factor in the nutrition and development of the child. Because negative emotions and anxiety during the feeding of the child may affect the function of the mother's cerebral cortex, hypothalamus, and pituitary gland, reduce the secretion of prolactin and oxytocin and finally reduce the breast milk secretion [34, 37].

Fallon et al. (2016) showed that women who have symptoms of postpartum anxiety are less likely to have exclusive breastfeeding, and it is more likely that they stop breastfeeding early and use formula [16]. Fallon et al., (2018), in another study using the PSAS, identified that higher levels of postpartum anxiety are associated with a lower likelihood of exclusive breastfeeding and breastfeeding at any rate in the first six months postpartum. PSAS scores were significantly associated with infant feeding behaviors. As a result, compared to the general anxiety and depression tools, the PSAS tool was a stronger predictor of infant feeding outcomes and behaviors [38].

Marital relation is another factor which is discussed in most studies. Though pregnancy is a physiological process, the physical changes that occur may increase the need for support felt by women [39]. Odinka et al. (2018) showed that women with postpartum anxiety and depression were 22% less satisfied with their married life [40]. Another longitudinal study on the relationship between marital life quality and postpartum depression and anxiety during the third trimester of pregnancy and four to six months after delivery showed that variables of marital relationship and marital satisfaction, postpartum depression levels and marital satisfaction significantly predicted postpartum anxiety levels. As a result, the quality of marital relationships should attract physicians' attention in the perinatal period. Providing psychological interventions to improve the performance of marital relationships may help prevent psychological distress in mothers [41].

Considering the crisis, we are currently facing and the unfortunate consequences of postpartum anxiety that has been neglected, and since there is no specific tool for measuring postpartum anxiety in crises, the importance of having a specific tool to measure postpartum anxiety increases in critical times.

### Strengths and limitations

The strengths of the study are randomly selecting samples from among women who have given birth, including women with a history of normal vaginal delivery and cesarean section, and conducting psychometric tests of the PSAS-IR-RSF-C scale for the first time in Iran. Conducting CFA and EFA on the same dataset was a limitation of the present study. Also, the possibility of potential bias due to the tendency to give socially desirable responses with self-reported measures, and placement of two items in two sub-dimension of nutrition and growth anxieties, and marital relationship anxieties are other limitations of the present study.

## Conclusions

Since the recent global health crisis has severely affected the mental health of pregnant women. In postpartum period, women will experience many problems in the field of mental health, which makes it necessary to have a short and specific tool for this period. Considering that this tool is valid and reliable for assessing postpartum anxiety for global crisis in Iranian postpartum women, health care providers can use it to evaluate the state of mental health in the postpartum period.

## Data Availability

The datasets generated and/or analysed during the current study are not publicly available due to limitations of ethical approval involving the patient data and anonymity but are available from the corresponding author on reasonable request.

## Abbreviations

PPA: Postpartum anxiety
PSAS-IR-RSF-C: Postpartum Specific Anxiety Scale – Research Short Form – for global Crises
EFA: Exploratory factor analysis
CFA: Confirmatory factor analysis
ICC: Intraclass Correlation Coefficient
SD: standard deviation
CVI: Content validity index
CVR: Content validity ratio
KMO: Kaiser–Meyer–Olkin
RMSEA: Root mean squared error of approximation
CFI: Comparative fit index
TLI: Tucker–Lewis index
